# Rp-HPLC Determination of Quercetin in a Novel D-α-Tocopherol Polyethylene Glycol 1000 Succinate Based SNEDDS Formulation: Pharmacokinetics in Rat Plasma

**DOI:** 10.3390/molecules26051435

**Published:** 2021-03-06

**Authors:** Osama A. A. Ahmed, Hany M. El-Bassossy, Heba M. El-Sayed, Soad S. Abd El-Hay

**Affiliations:** 1 Department of Pharmaceutics, Faculty of Pharmacy, King Abdulaziz University, Jeddah 21589, Saudi Arabia; 2 Center of Excellence for Drug Research and Pharmaceutical Industries, King Abdulaziz University, Jeddah 21589, Saudi Arabia; 3 Mohamed Saeed Tamer Chair for Pharmaceutical Industries, King Abdulaziz University, Jeddah 21589, Saudi Arabia; 4 Department of Pharmacology and Toxicology, Faculty of Pharmacy, Zagazig University, Zagazig 44519, Egypt; helbassossy@pharmacy.zu.edu.eg; 5 Department of Analytical Chemistry, Faculty of Pharmacy, Zagazig University, Zagazig 44519, Egypt; heba328@yahoo.com (H.M.E.-S.); soadselem@gmail.com (S.S.A.E.-H.)

**Keywords:** quercetin, TPGS, SNEDDS formulation, RP-HPLC, pharmacokinetics study, rat plasma

## Abstract

Despite its proven efficacy in diverse metabolic disorders, quercetin (QU) for clinical use is still limited because of its low bioavailability. D-α-Tocopherol polyethylene glycol 1000 succinate (TPGS) is approved as a safe pharmaceutical adjuvant with marked antioxidant and anti-inflammatory activities. In the current study, several QU-loaded self-nanoemulsifying drug delivery systems (SNEDDS) were investigated to improve QU bioavailability. A reversed phase high performance liquid chromatography (RP-HPLC) method was developed, for the first time, as a simple and sensitive technique for pharmacokinetic studies of QU in the presence of TPGS SNEDDS formula in rat plasma. The analyses were performed on a Xterra C_18_ column (4.6 × 100 mm, 5 µm) and UV detection at 280 nm. The analytes were separated by a gradient system of methanol and phosphate buffer of pH 3. The developed RP-HPLC method showed low limit of detection (LODs) of 7.65 and 22.09 ng/mL and LOQs of 23.19 and 66.96 ng/mL for QU and TPGS, respectively, which allowed their determination in real rat plasma samples. The method was linear over a wide range, (30–10,000) and (100–10,000) ng/mL for QU and TPGS, respectively. The selected SNEDDS formula, containing 50% *w*/*w* TPGS, 30% polyethylene glycol 200 (PEG 200), and 20% *w*/*w* pumpkin seed oil (PSO), showed a globule size of 320 nm and −28.6 mV zeta potential. Results of the pharmacokinetic studies showed 149.8% improvement in bioavailability of QU in SNEDDS relative to its suspension. The developed HPLC method proved to be simple and sensitive for QU and TPGS simultaneous determination in rat plasma after oral administration of the new SNEDDS formula.

## 1. Introduction

**Figure 1:**
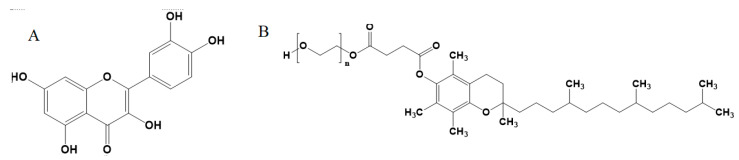
Chemical structure of quercetin, **A**, and D-α-Tocopherol polyethylene glycol 1000 succinate (TBGS), **B**.

Quercetin (QU), 3,3′,4′,5,7-pentahydroxyflavone (Figure 1A), is a natural substance belonging to the flavonoids family present in food including chamomile, honey, and passionflower [1]. QU confers diverse health benefits like anti-inflammatory and antioxidant activities. Such effects are very important for prevention and even treatments of a wide array of diseases and disorders like diabetes [2], metabolic syndrome, and cardiovascular diseases [3]. However, low aqueous solubility and bioavailability is considered a barrier for the clinical application of QU [4].

The diverse health benefits of QU draw the attention of scientific community to improve QU delivery through a colon-targeted system [5]. Additionally, different nano-based QU delivery systems were utilized [6,7,8,9,10] for reviews [11,12]. The different delivery systems which formulated to enhance QU bioavailability such as nanoparticles, nano emulsion, and solid dispersion have limitations concerning their affordability, safety, efficacy, and stability. Therefore, a new formulation was needed to overcome these drawbacks.

The ability of self-nanoemulsifying drug delivery systems (SNEDDS) to improve lipophilic drugs’ oral bioavailability has drawn attention to this field. The enhancement in bioavailability is related to the spontaneously formed emulsion in the aqueous gastrointestinal tract medium that improves drugs dissolution. The bioavailability-enhancing property is also associated with a reduction of the first pass effect and hence a reduction in liver drug metabolism [13,14,15].

D-α-Tocopherol polyethylene glycol 1000 succinate, TPGS (Figure 1B), is a vitamin E derivative characterized by its aqueous solubility. It is synthesized by tocopherol acid succinate esterification with polyethylene glycol 1000 (PEG 1000). The amphiphilic character of TPGS enhances drug solubility [16]. TPGS was approved by The United States Food and Drug Administration (US FDA) as a safe pharmaceutical formulation adjuvant [17]. Being a derivative of vitamin E, TPGS has marked antioxidant and anti-inflammatory activities [16]. TPGS is useful as a carrier molecule for drug delivery. TPGS also exerts intrinsic therapeutic effects with possible synergistic interactions with formulated active ingredients. TPGS was determined by HPLC-UV methods in its commercially available products or biological fluids [18,19].

Different HPLC techniques described determination of QU alone or in combination with other flavonoids or related compounds in spiked or real human or rat plasma [20,21,22,23,24,25,26]. However, flaws of the reported methods, the expensive, inaccessible liquid chromatography-mass spectrometry (LC-MS) methods and the time-consuming HPLC-UV ones, were noticed.

The team of this work have developed for the first time, to the best of our knowledge, a simple, sensitive, and cost-effective HPLC-UV method for determination of QU in the presence of TPGS in rat plasma to study the pharmacokinetic properties of the new QU-loaded TPGS-based SNEDDS.

## 2. Results and Discussion

The developed HPLC method was sensitive to determine quercetin in plasma after administration of the multicomponent novel SNEDDS nano formulation. Therefore, it enabled us to carry out the pharmacokinetic study and evaluate the current novel formulation. The reported LC-MS methods showed a low LOQ but required sophisticated and expensive techniques, which are not easily available in most laboratories. In comparison with the reported HPLC-UV methods, our proposed method offers a lower LOD (7.65 vs. 16.67–200 ng/mL of the reported methods) as well as a shorter runtime. In addition, none of the reported methods determined Qu and TPGS simultaneously.

### 2.1. Method Development

**Figure 2:**
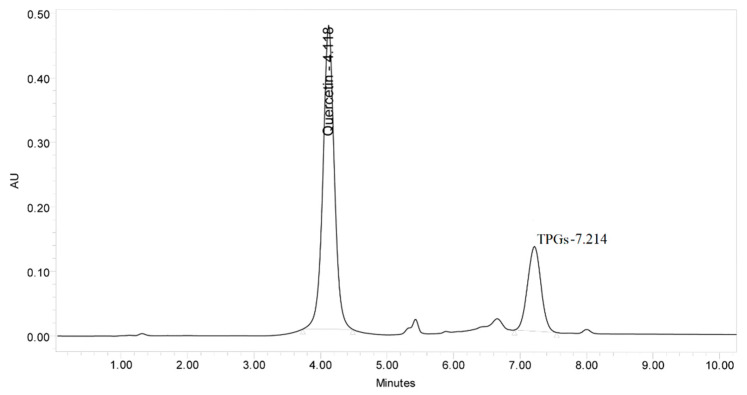
Representative chromatogram of a standard mix of 100 µg/mL quercetin and D-α-Tocopherol polyethylene glycol 1000 succinate (TPGS) using gradient elution of methanol (A) and phosphate buffer of pH 3 ± 0.1 (B), 50/50 (A/B, *v*/*v*) for 5 min, 98/2 (A/B, *v*/*v*) for 5 min, and 50/50 (A/B, *v*/*v*) for 5 min at flow rate 1 mL/min, 280 nm, and at 25 ± 5 °C.

Separation of QU and TPGS was achieved by a simple RP-HPLC method either in pure form or in rat plasma. The pharmacokinetic parameters of QU in the new QU-loaded TPGS-based SNEDDS formulation were successfully studied by the applied method. Different chromatographic conditions were investigated to accomplish the best separation and sensitivity. Five different columns, namely Zorbax C_18_ column (150 × 4.6 mm, 5 µm), Equisil BDS, C_18_, (150 × 4.6 mm, 5 µm), Reprosil Gold, C_18_, (250 × 4.6 mm, 5 µm), Chromollith^®^, C_18_, (100 × 4.6 mm, 5 µm), and Xterra C_18_ column (100 × 4.6 mm, 5 µm) were used as the stationary phases. The best separation within a reasonable time with no interference from plasma peaks was achieved using the Xterra C_18_ column. Different temperatures (30–45 °C) were studied, but no significant differences were observed. The examined UV detection wavelengths ranged from 210 to 370 nm. Both the isocratic and gradient elution modes were tried using different percentages of methanol or acetonitrile (35% to 98%) with water or a phosphate buffer of different pH (3–5) to get the best separation conditions. Isocratic elution failed in good separation of TPGS and gave poorly shaped broad peaks, especially when using the Reprosil Gold C_18_ column. The optimum mobile phase consisted of methanol (A) and phosphate buffer of pH 3 ± 0.1 (B). Gradient elution of 50/50 (A/B, *v*/*v*) for 5 min, 98/2 (A/B, *v*/*v*) for 5 min, and 50/50 (A/B, *v*/*v*) for 5 min at a flow rate of 1 mL/min, and the total run was carried out at 25 ± 5 °C with UV detection at 280 nm. The HPLC chromatogram of QU and TPGS under the best conditions is shown in (Figure 2).

Protein precipitation was applied for sample clean-up as the simplest and most time saving technique. Methanol, acetonitrile, methanol: 0.1% formic acid in water, 60:40, *v*/*v* [22] were tried as precipitating agents in a ratio of 1:5 (plasma:organic precipitant) or by liquid–liquid extraction using ethyl acetate [4]. Ethyl acetate achieved maximum sensitivity for both analytes. Sample dilution is the main disadvantage [27]. This was overcome by evaporation to dryness, then reconstitution in 100 µL methanol.

### 2.2. Validation of the HPLC Method

#### 2.2.1. Specificity

**Figure 3:**
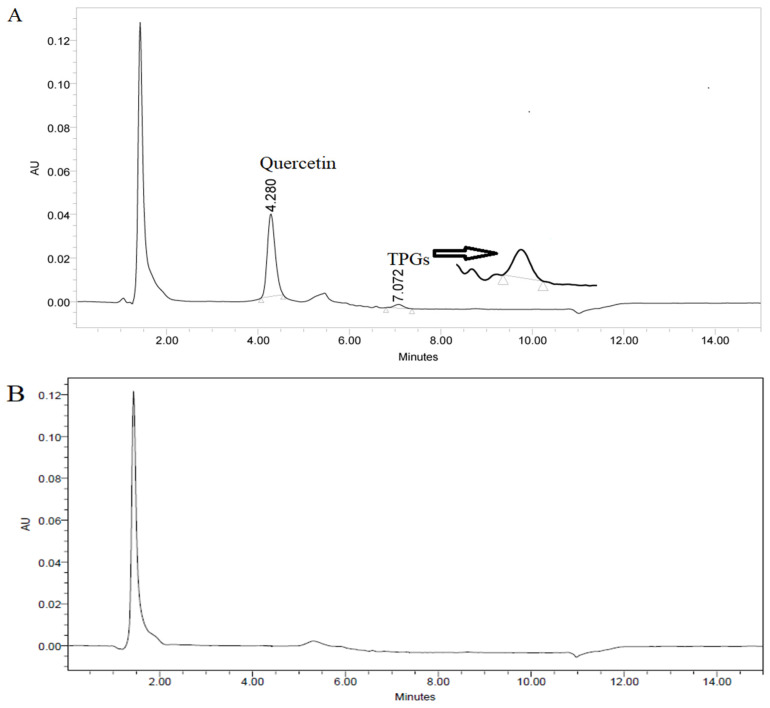
Representative chromatogram of 10 µg/mL quercetin and D-α-Tocopherol polyethylene glycol 1000 succinate (TPGS) in rat plasma under the optimized conditions, **A** against the blank plasma, **B**.

Method selectivity was confirmed by analyzing the extracted spiked plasma. Comparing the chromatogram of QU in rat plasma samples (Figure 3A) with those of a blank sample (Figure 3B) showed no interfering peaks from plasma around the retention times of QU and TPGS. In addition, the developed method was effectively applied for determination of QU and TPGS in rat plasma after the novel SNEDDS formulation was administered orally.

#### 2.2.2. System Suitability

Method system suitability was studied by analyzing retention time, capacity factor, symmetry, resolution, and number of theoretical plates, as shown in Table 1.

#### 2.2.3. Linearity, LOD, and LOQ

**Figure 4:**
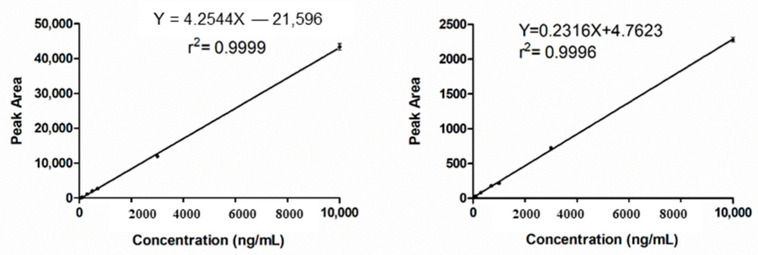
Calibration curves for Quercetin, **A**, and D-α-Tocopherol polyethylene glycol 1000 succinate (TPGS), **B**.

Calibration curves were constructed by analyzing a series of calibration standards in the concentration ranges of (30–10,000), (100–10,000) ng/mL for QU and TPGS, respectively. The resulting peak area was plotted against the corresponding concentration (Figure 4). The calibration curves showed a high value of the determination coefficient; r^2^ ≥ 0.9996 (Table 2).

Recovery percentages for the analytes show acceptable values. They were within ± 8% of their theoretical values (Table 3). The high sensitivity of our method represented by the low LOD and LOQ values for both Qu and TPGS enables their determination in real plasma samples with high reliability (Table 2).

#### 2.2.4. Accuracy and Precision

QC samples were analyzed as under Section 3.3. Accuracy studies were used to determine the recoveries of plasma samples spiked with studied analytes. Method accuracy was confirmed by the acceptable % recoveries and small relative error (RE%) as shown in Table 4.

Additionally, the intra-day and inter-day precision of the assay was performed using the same QC samples, which were injected in three replicates on the same day and on three different days. The low relative standard deviation values (RSD%) indicated the high precision of the method (Table 4).

#### 2.2.5. Matrix Effect (ME)

The reported studies revealed the extensive binding of QU to human plasma proteins; the protein binding percentage is 99.4 ± 0.1% [28]. Matrix components effect on QU quantitation was investigated by spiked plasma sample analysis. ME (%) was computed using the peak area of QU extracted from spiked plasma samples (Ai) ratio with those of the pure standard solutions (Ar), according to the equation ME (%) = Ai/Ar × 100. The calculated ME% for Qu was 98.93 ± 4.91% with no significant interference from matrix components.

#### 2.2.6. Stability

A stability assay comprising freeze–thaw stability for matrix-based samples was conducted using QC samples. RSD of the mean test responses was within 3% in all stability tests. No degradation was observed through three freeze (−80 °C)–thaw (room temperature) cycles. In addition, plasma samples of QU were stable for at least 15 days at −80 °C, respectively.

### 2.3. Formulation Studies

**Figure 5:**
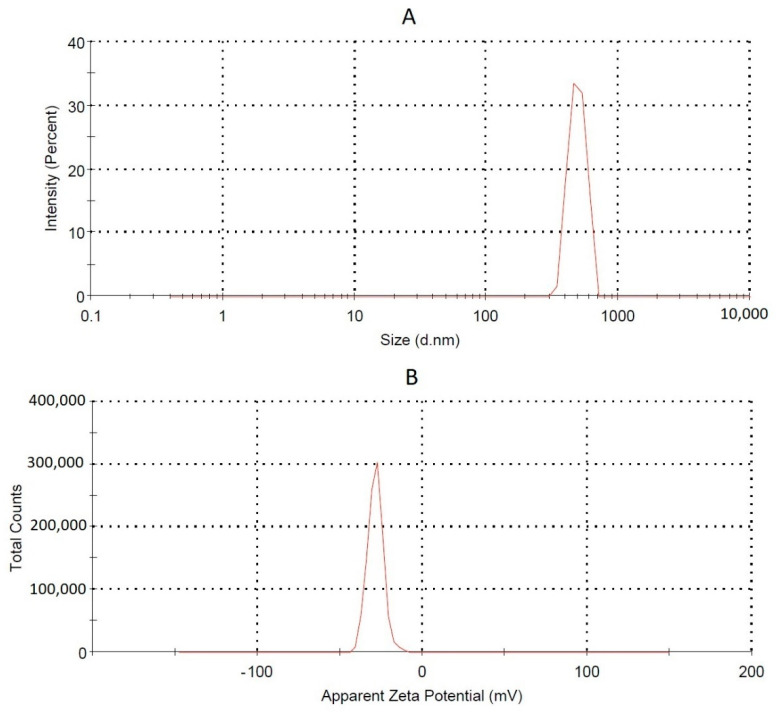
Globule size, **A**, and zeta potential, **B**, of the selected SNEDDS formulation (F5).

Different SNEDDS formulations were investigated as indicated in Table 5. The prepared SNEDDS formulations showed variations in globule size and zeta potential. The polydispersity index (PDI) ranged from 0.23 (F3) to 0.7 (F2), which indicates variation in globule size distribution according to formula composition, Table 5. The results revealed that at a high oil concentration, the globule size exceeded the nano-range (>1000 nm), while at a low oil concentration, the globule size decreased to <100 nm. Although SNEDDS formula F4 showed the lowest globule size, formula F5 was selected based on the highest possible PSO and TPGS concentrations with the lowest/moderate globule size (Table 5). The prepared formula was selected for in-vivo studies. The prepared QU-SNEDDS formulation showed a globule size of 320 nm (Figure 5A) and zeta potential of −28.6 mV (Figure 5B). The nano-pharmaceutical formula of QU SNEDSS consists of safe ingredients forming a nano-emulsion. The PSO utilized is a natural oil, and TPGS and PEG are approved by the FDA. All ingredients of the formulation have a reported antioxidant activity [16,29,30]. The antioxidant, solubilizer, and permeation enhancement activity of TPGS can improve the bioavailability of loaded hydrophobic drugs [14,31]. TPGS has a P-glycoprotein (P-gp) inhibition activity that could augment the efficacy of QU [32].

### 2.4. Pharmacokinetic Study in Rats

**Figure 6:**
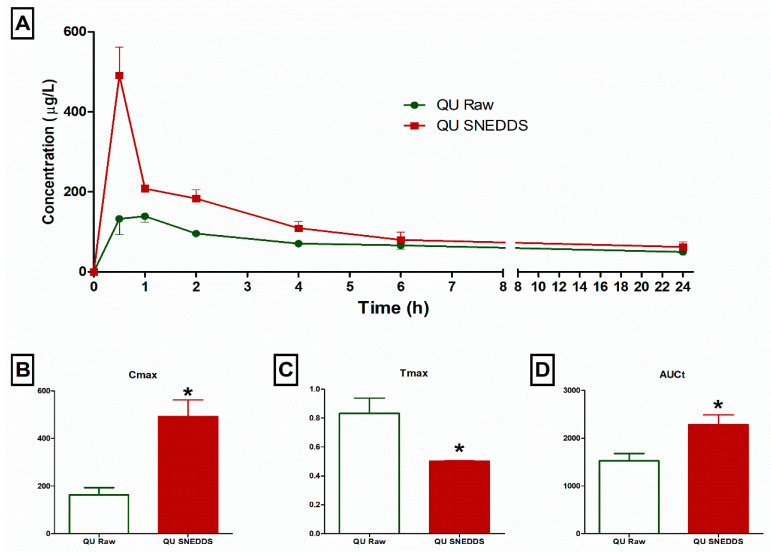
Plasma concentration of quercetin (QU) within 24 h after administration of QU SNEDDS formula and QU suspension, **A**. Comparison of C_max_, **B**, T_max_, **C**, and AUCt, **D**. Data presented as mean ± SEM, *n* = 7 animals, * Significantly different (*p* < 0.05).

The plasma QU concentration time curve for the QU suspension compared with QU SNEDDS formula within a 24 h period is displayed in (Figure 6). The results revealed enhanced QU delivery from the selected QU SNEDDS formula compared to a QU suspension (Figure 6A). The pharmacokinetic results revealed significant (*p* < 0.05) improvement in C_max_, T_max_, and AUCt results for QU SNEDDS formula when compared to QU suspension results. QU SNEDDS formula showed elevated C_max_ value of 491.3 ± 172.2 µg/L compared to QU suspension value of 163.2 ± 74 µg/L (Figure 6B). Additionally, the QU SNEDDS formula reduced T_max_ to 0.5 ± 0.0 h compared to 0.83 ± 0.26 h (Figure 6C). In addition, the AUCt data were significantly improved with 2286 ± 500.1 µg h/L and 1525.7 ± 378.8 for QU SNEDDS formula and QU suspension, respectively (Figure 6D). The plasma and pharmacokinetics findings revealed significant improvements in QU bioavailability on administration of QU SNEDDS formula by 149.8% relative to that of QU suspension.

The improvement in pharmacokinetic results of the QU-SNEDDS formula could be a result of SNEDDS ability to enhance the permeability of the gut membrane for the transport of oily compounds [33]. The instant self-emulsification offers QU in small globule solubilized form that massively increases the surface area (space) for QU absorption in the gastrointestinal tract [34]. Compared to regular formulations, QU SNEDDS offers a more stable and efficient formulation for improving the absorption rate and extent [31,35,36,37].

## 3. Materials and Methods

### 3.1. Chemicals and Reagents

QU (purity ≥95), TPGS (BioXtra) and polyethylene glycol 200 (PEG 200) were purchased from Sigma-Aldrich (St. Louis, MI, USA). HPLC grade acetonitrile, methanol, formic acid, and ethyl acetate were provided by Lichrosolv (Darmstadt, Germany). A double distilled water system from Milli-Q^®^ (Burlington, MA, United States) was used.

### 3.2. Apparatus and Analytical Conditions

Chromatographic separation was done on a Waters LC 2695 system (Milford, MA, USA) with a quaternary, low-pressure mixing pump, inline vacuum degassing, and a Waters 996 PDA detector (200–600 nm). Data collection and analysis were done by Empower software (version 3, Waters Corporation, Milford, MA, USA). Mixture separation was achieved using an Xterra C18 column (4.6 × 100 mm, 5 µm, Waters, Milford, MA, USA). The column temperature was maintained at 25 ± 5 °C. A mobile phase composed of methanol (A) and phosphate buffer of pH 3 ± 0.1 (B) was used. The analytes were separated by the gradient elution of A/B (*v*/*v*) as follows: 50/50 for 5 min, 98/2 for 5 min, then 50/50 for 5 min; the total run time was 15 min. The injection volume was 10 µL and UV detection was done at 280 nm.

### 3.3. Preparation of Stock and Working Solutions

QU and TPGS stock solutions were prepared as 1 mg/mL in methanol. The working solutions of both analytes were prepared by dilution with methanol at concentrations of (300–100,000), (1000–100,000) ng/mL for QU and TPGS, respectively. The calibration solutions in the concentration ranges of (30–10,000), (100–10,000) ng/mL for QU and TPGS, respectively, were prepared by mixing 10 µL of working solutions with 90 µL of rat blank plasma. Then, 500 µL of ethyl acetate was added, vortex mixed for 3 min, and the mixture was set aside for 10 min then centrifuged at 4 °C at 15,000 rpm for 15 min. The upper organic phase was transferred to another Eppendorf tube and dried under nitrogen at room temperature (25 °C), reconstituted in 100 μL of methanol, vortex mixed for 2 min, and then transferred to autosampler vial. Then, 10 µL was injected. A blank was prepared using 100 µL rat blank plasma, then the procedure was completed as before. The quality control samples (QC) samples at low, medium, and high concentrations (100, 3000, and 10,000 ng/mL) for QU and TPGS were injected three times in the same day and for three different consecutive days.

### 3.4. Sample Preparation

Rat plasma (100 µL) was added to a 1.5 mL Eppendorf tube. Then, the procedure was completed as under Section 3.3.

### 3.5. Method Validation

The developed method’s specificity, linearity, limit of detection (LOD), limit of quantification (LOQ), matrix effect (ME), inter-day and intra-day precision, accuracy, and stability were verified according to the US FDA guidelines for bioanalytical method validation [38].

### 3.6. Formulation Studies

Different concentrations of PSO, TPGS, and PEG 200 were utilized in the formulation of QU SNEDDS preparations. QU loading in the prepared SNEDDS formulations was fixed at 25 mg/1 g SNEDDS formula, and the components of the SNEDDS formula (PSO, TPGS and PEG 200) were added up to 100%. QU-SNEDDS formula was prepared as previously reported with slight modifications [39]. QU (25 mg) was added to the specified amount of PSO and PEG 200 indicated in Table 5 and mixed. TPGS was melted at 40 °C and added to the mixture that kept at 40 °C. The mixture was vortexed for 3 min and subjected to probe sonication for 1 min. The percentage of SNEDDS components were kept at 100% (1 g total weight).

#### QU-SNEDDS Globule Size and Zeta Potential Determination

Size and zeta potential of the QU-SNEDDS formulations as well as PDI were investigated by Nano-ZSP, Marlvern Instrument, Worcestershire, UK, using a dynamic light scattering technique; 100 μL of each QU-loaded SNEDDS was diluted with 10 mL of 0.1 N HCl then vortexed for 60 s and then analyzed.

### 3.7. Application to Pharmacokinetic Study in Rats

#### 3.7.1. Experimental Animals

Male Wistar (6–8-week-old, 180–200 g) rats were obtained from Faculty of Pharmacy animal house (Zagazig University, Egypt). Transparent polypropylene cages were utilized (four rats in each cage) with access to standard rodent pellets and purified water. Appropriate animal housing conditions of rotating 12 h light and dark, 22 ± 3 °C, 50–60% relative humidity, and suitable ventilation were applied. The protocol for the experimental work was approved by the Institutional Animal Care and Use Committee at Zagazig University (ZU-IACUC, approval number: ZU-IACUC/3/F/122/2020).

#### 3.7.2. Design of Pharmacokinetic Study

The animals were randomly divided into three experimental groups (seven animals each): a standard QU group (Q) administered 25 mg/kg QU suspension; a nano formula vehicle group (V) that received the same volumes of SNEDDS ingredients including TPGS without QU; and the nano formula group (NQ) that was administered 25 mg/kg of QU in the form of a nano-pharmaceutical formulation. All preparations were administered once by oral gavage, then 0.5 mL blood was collected from the retro-orbital plexus vein in heparinized tubes under light ether anesthesia after 0.5, 1, 2, 4, 6, and 24 h after administration for determination of QU by HPLC. Blood samples were then centrifugated at 10,000 rpm for 10 min at 4 °C to separate plasma. Samples were kept at −80 °C until analysis.

#### 3.7.3. Pharmacokinetic Analysis

Analysis of plasma concentration versus time data were utilized for the analysis of pharmacokinetic parameters. The calculated pharmacokinetic parameters were maximum plasma concentration (C_max_), time to reach C_max_ (T_max_), and area under plasma concentration–time curve (AUCt).

#### 3.7.4. Statistical Analysis

The analysis was carried using Graph Pad Prism (version 5.0, Graph Pad, San Diego, CA, USA) and the pharmacokinetics results were represented as mean ± SEM. Statistical comparison between QU raw and QU SNEDDS was done using unpaired Student t test, and the *p* < 0.05 value was taken into consideration as significant.

## 4. Conclusions

QU has proven its health benefits in prevention and even treatments of a wide array of diseases and disorders such as diabetes, metabolic syndrome, and cardiovascular dis-eases. However, its clinical use remains limited because of its low aqueous solubility and bioavailability. Among the tested several QU-loaded self-nanoemulsifying drug delivery systems (SNEDDS), only D-α-Tocopherol polyethylene glycol 1000 succinate based SNEDDS showed a globule size of 320 nm and −28.6 mV zeta potential. The developed simple but sensitive RP-HPLC method enabled simultaneous determination of QU and TPGS in rat plasma after oral administration of the novel SNEDD formula. Application of the proposed RP-HPLC method to pharmacokinetic studies revealed the improvement in QU bioavailability by 149.8% relative to that of QU suspension. Therefore, the formulated QU-SNEDDS can be considered as an effective formulation to overcome the poor bioavailability of QU.

## 5. Patents

This SNEDDS preparation of quercetin is protected under USSN 16/741,826, United States Patent and Trademark Office (USPTO).

## Figures and Tables

**Table 1:** System suitability parameters of the proposed HPLC-UV method in spiked rat plasma.

Parameter	Quercetin	TPGS
Retention time (Rt)	4.11 ± 0.02	7.21 ± 0.14
Capacity Factor (k’)	1.74	3.81
Resolution (Rs)	-	9.26
Selectivity (α)	-	2.18
Symmetry factor	1.03	1.034
No of theoretical plates (N) (plates/m)	2.91 × 10^3^	5.96 × 10^3^

**Table 2:** Validation parameters of the proposed HPLC-UV method in spiked rat plasma.

Parameter	Quercetin	TPGS
Linearity range (ng/mL)	30–10,000	100–10,000
Regression equation parameters Y= ax + b		
Slope (a)	4.254	0.231
Intercept (b)	−215.96	4.763
Determination Coefficient (r^2^)	0.9999	0.9996
Limit of detection LOD (ng/mL)	7.65	22.09
Limit of quantitation LOQ (ng/mL)	23.19	66.96

**Table 3:** Assay results for the determination of the studied analytes in spiked rat plasma.

Quercetin			TPGs		
Added Concentration (ng/mL)	Found Concentration (ng/mL) *	Recovery (%)	Added (ng/mL)	Found (ng/mL) *	Recovery (%)
100	102.97	102.97	100	96.01	96.02
300	308.60	102.87	300	308.32	103.25
500	521.30	104.26	700	750.16	107.35
700	696.08	99.44	3000	3096.73	103.24
3000	2926.62	97.55	10,000	9980.93	99.79
10,000	10,020.94	100.21			
**Mean**		101.22			101.93
**SD**		2.56			4.25
**RSD**		2.525			4.170

* Average of three determinations.

**Table 4:** Accuracy and precision evaluation of the proposed method.

Analyte	Concentration (ng/mL Plasma)	Accuracy		Precision	
		Mean% Recovery*	RE%	Intra-Day RSD%	Inter-Day RSD%
**Quercetin**	100	100.27	0.27	0.778	0.894
	3000	100.99	0.99	0.346	1.782
	10,000	101.22	1.22	0.789	0.304
**TPGS**	100	100.91	0.91	1.542	2.594
	3000	99.56	−0.43	1.423	2.633
	10,000	99.81	−0.19	0.820	0.300

* Mean recovery for three determinations.

**Table 5:** SNEDDS formulations and their observed globule size.

Formula #	PSO	TPGS	PEG 200	Size (nm) ± SD	PDI ± SD	Zeta Potential mV ± SD
**F1**	0.1	0.3	0.6	165.3 ± 26.1	0.29± 0.02	−21.2 ± 4.3
**F2**	0.4	0.3	0.3	>1000	0.7 ± 0.2	−27.4 ± 6.8
**F3**	0.2	0.4	0.4	362 ± 29.0	0.23 ± 0.05	−29.3 ± 3.8
**F4**	0.1	0.5	0.4	82.6 ± 25.3	0.35 ± 0.03	−26.2 ± 5.2
**F5**	0.2	0.5	0.3	320 ± 34.3	0.37 ± 0.07	−28.6 ± 4.1
**F6**	0.3	0.3	0.4	490 ± 59.8	0.42 ± 0.03	−30.2 ± 3.8

## Data Availability

Data available on request.
